# Exploring the Role of the Plant Actin Cytoskeleton: From Signaling to Cellular Functions

**DOI:** 10.3390/ijms242015480

**Published:** 2023-10-23

**Authors:** Guoqiang Yuan, Huanhuan Gao, Tao Yang

**Affiliations:** Ministry of Education Key Laboratory of Cell Activities and Stress Adaptations, School of Life Sciences, Lanzhou University, Lanzhou 730000, China; yuangq19@lzu.edu.cn (G.Y.); gaohh@lzu.edu.cn (H.G.)

**Keywords:** actin cytoskeleton, ARPs, ABPs, signal crosstalk, biological functions, microtubules, plant development

## Abstract

The plant actin cytoskeleton is characterized by the basic properties of dynamic array, which plays a central role in numerous conserved processes that are required for diverse cellular functions. Here, we focus on how actins and actin-related proteins (ARPs), which represent two classical branches of a greatly diverse superfamily of ATPases, are involved in fundamental functions underlying signal regulation of plant growth and development. Moreover, we review the structure, assembly dynamics, and biological functions of filamentous actin (F-actin) from a molecular perspective. The various accessory proteins known as actin-binding proteins (ABPs) partner with F-actin to finely tune actin dynamics, often in response to various cell signaling pathways. Our understanding of the significance of the actin cytoskeleton in vital cellular activities has been furthered by comparison of conserved functions of actin filaments across different species combined with advanced microscopic techniques and experimental methods. We discuss the current model of the plant actin cytoskeleton, followed by examples of the signaling mechanisms under the supervision of F-actin related to cell morphogenesis, polar growth, and cytoplasmic streaming. Determination of the theoretical basis of how the cytoskeleton works is important in itself and is beneficial to future applications aimed at improving crop biomass and production efficiency.

## 1. Introduction

The optimization of cellular architecture is one of the most efficient means to advance crop productivity and stress tolerance. While plant cell growth and morphology are modulated by a number of factors, the actin cytoskeleton plays a decisive role in almost all plant developmental processes, including cargo trafficking, cellular motility, apical growth, cell wall formation, and cytoplasmic streaming [1,2,3,4,5,6]. In the cytoplasm, actin has different physiological functions depending on its form, as a monomer, oligomer, or polymer or as a complex with actin-binding proteins (ABPs). Polymeric and non-polymeric forms of actin maintain a dynamic balance in the cell. Actin molecules come together into a filament, a double helix polymer with a head-to-tail orientation that gives the filament a +/− molecular polarity. This polarity is crucial to the mechanism of actin assembly, with the barbed (or +) end of a filament always elongating much faster than the pointed (or –) end. Shortly after each actin monomer assembles into a filament, its bound ATP is hydrolyzed into ADP, such that the filament is a linear mix of ATP-actin and then ADP-actin. ATP-actin that associates at the barbed end and ADP-actin that dissociates from the pointed end are affected by ATP hydrolysis and phosphate dissociation. The overall mechanism at steady state denotes a dynamic equilibrium known as “treadmilling” [7,8]. The cell monitors both temporal and spatial actin dynamics, such as actin filament nucleation, movement, assembly, and disassembly. As such a basic part of cellular function, it is necessary to understand the intriguing roles of actin proteins in various cellular processes.

The actin-related proteins (ARPs) were discovered in eukaryotes in the 1990s. The ARPs are classified into various classes or subfamilies, which are extremely conserved across a wide range of eukaryotes, from yeast to plants and humans [9]. The amino acid sequence of ARPs differs by 20% to 60% compared to the canonical actin [10]. Nevertheless, the expression patterns of ARP genes do not correlate strongly with those observed for either actins or ABPs [11]. Actually, ARP classes assemble with other proteins to form stable hetero-multimeric protein complexes [12,13,14], so it is important to take into account how ARPs function biologically in light of their roles as parts of larger macromolecular machinery.

ABPs have evolved as partners that facilitate and manipulate the formation and turnover of actin filaments [2,7,8]. Genetic or physical interactions between actin and different ABPs can be placed into various signaling networks that participate in specific plant morphogenetic pathways that are supervised by the actin cytoskeleton. A combination of approaches has been used to predict the impact of ABPs on actin dynamics so that a deeper understanding of actin-mediated functions in plants can be achieved [15]. Beyond that, an increasing number of ABPs and microtubule-associated proteins (MAPs) have been identified as indispensable elements for sensing environmental signals and for the coordinated regulation of cytoskeleton reorganization [16,17,18,19]. Whether the functions of plant and animal ABPs are evolutionarily conserved has been the thrust of research in the field for a long time.

In this review, we summarize recent reports that have uncovered the functions of actin and ARPs during the growth and development of plants and highlight critical new genetic and biochemical evidence that plant ABPs coordinate filamentous actin (F-actin) formation to perform different functions. Additionally, these complex arrangements reveal crosstalk between the actin cytoskeleton and regulatory proteins within signaling networks in response to environmental change. Despite recent progress, research on actin in plants is rather fragmentary in comparison to our understanding of the animal actin cytoskeleton. We hope that this review provides a serviceable update on the functional properties of actin proteins in multiple plant species.

## 2. Identification and Annotation of Arabidopsis Actin Family

Actin, as a cytoskeletal protein, is encoded by a relatively diverse and ancient family of genes whose phylogeny began with the emergence of vascular plants [20]. We focus this part of the review on differential expression of genes within the actin family and their different functions as selective forces that preserve divergent members.

### 2.1. Tissue-Specific Expression of Arabidopsis Actin Genes

**Figure 1:**
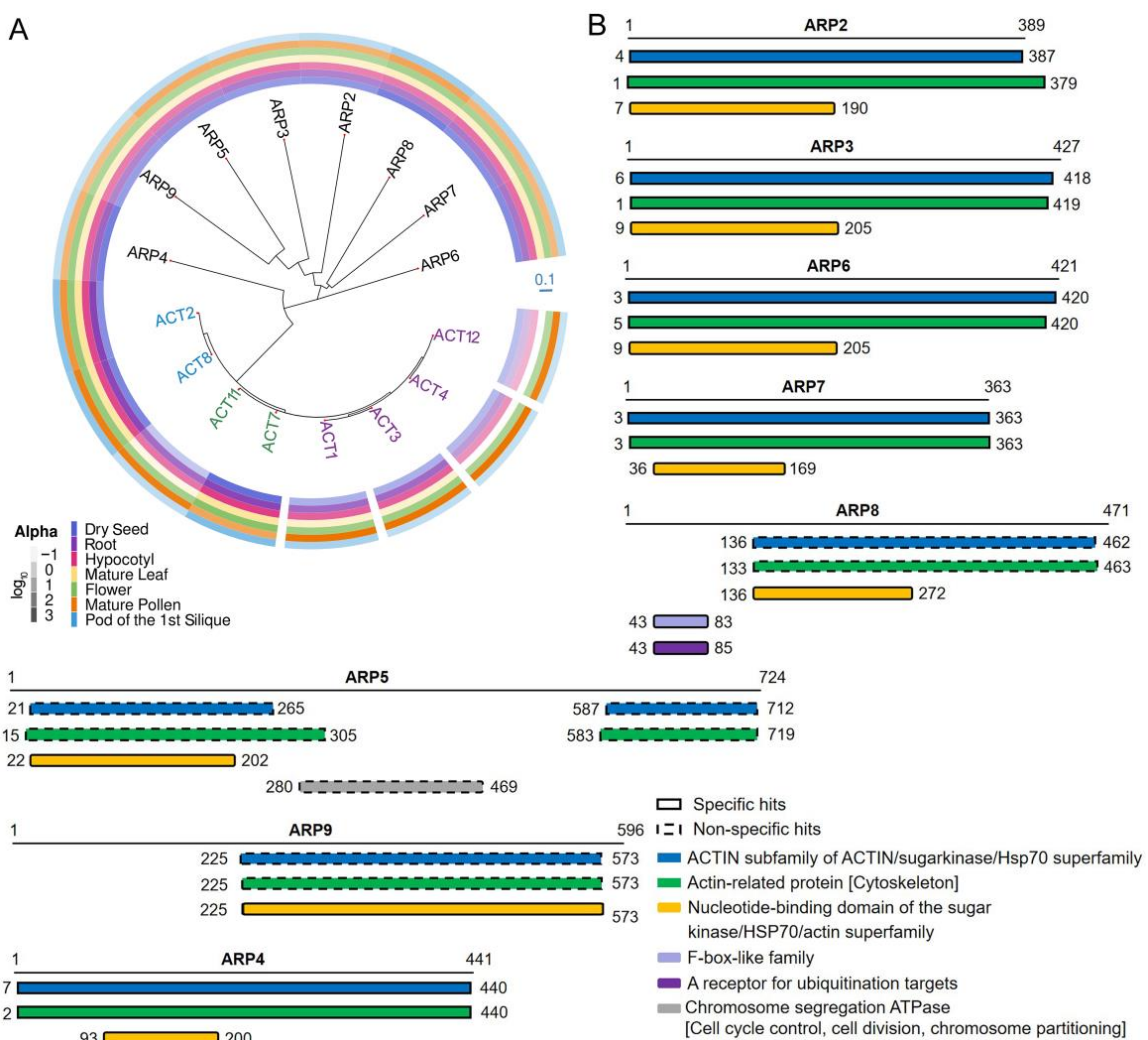
Homology analysis of actins and ACTIN-RELATED PROTEINs (ARPs). (**A**) Phylogenetic relationships of actin proteins and ARPs. The protein sequences of Arabidopsis actins (AtACT1, AtACT2, AtACT3, AtACT4, AtACT7, AtACT8, AtACT11, and AtACT12) and ARPs (AtARP2, AtARP3, AtARP4, AtARP5, AtARP6, AtARP7, AtARP8, and AtARP9) are compared in a neighbor-joining (NBJ) tree. The branch length in the NBJ tree relays the degree of sequence divergence or genetic distance. Therefore, compared with actins, the accelerated rate of divergence among Arabidopsis ARPs is greater throughout the course of evolution. The expression patterns in different organs (dry seed, root, hypocotyl, mature leaf, flower, mature pollen, and pod of the first silique) of the ARPs and actins are shown in the color bars, which display the log_10_ expression value acquired the integration platform ePlant. (ePlant: Visualizing and Exploring Multiple Levels of Data for Hypothesis Generation in Plant Biology, http://bar.utoronto.ca/eplant (accessed on 14 September 2021)). (**B**) The search for conserved domains in the ARP protein sequences from NCBI (National Center for Biotechnology Information, https://www.ncbi.nlm.nih.gov/cdd (accessed on 14 September 2021)) is shown.

There are ten actin genes in Arabidopsis, eight of which are strongly expressed at certain times and locations during plant development [21]. Vegetative actins (*ACT2*, *ACT7* and *ACT8*) are strongly expressed in the roots, stems, and leaves of germinating seedlings, young plants, and mature plants. In contrast, five other actin genes, namely *ACT1*, *ACT3*, *ACT4*, *ACT11*, and *ACT12*, are characterized by expression in pollen, ovules, and seeds [20]. Joint analysis of the phylogeny and tissue localization of the actin family proteins shows that the division of each member in the phylogenetic tree is distinct, while their gene expression patterns partially overlap (Figure 1A). The rate of divergence among actins is mild, with members of the actin family more closely related than members of the ARP family. This suggests that the evolution of respective regulatory elements with each protein may be more complex in comparison to that of their coding sequences. Given that the expression patterns of actin genes have remained consistent throughout lengthy evolutionary time periods, it is reasonable to consider selective constraint keeping the actin family so conserved.

### 2.2. Subcellular Localization of Actin

The subcellular localization of each actin protein will be related to its function. Prediction of the subcellular localization of the sequences highly homologous to Arabidopsis ACT1 in other species of plants has been completed on the crop proteins with annotated locations (cropPAL, https://croppal.org/ (accessed on 27 February 2022)). A query of the database showed that actins may be present in the nucleus, cytoplasm, and plastids (Table S1). We specifically list the predicted subcellular localizations of different actin isoforms in Arabidopsis, and the data from different sources highlight the cytoskeleton, nucleus, and cytosol as the main sites of actin accumulation (Table S2).

However, many non-specific interactions between actin and different kinds of binding partners have been reported, fueling debate about the function of actin-nucleoprotein binding. The functions of actin in the nucleus are better understood in mammalian cells compared to plant cells. Although it is not clear how much actin is normally present in the nucleus, a few studies have revealed that actin could shuttle in and out of the nucleus [22,23]. In mammalian cells, members of the actin family take part in a variety of activities in the nucleus in multifarious forms, including monomers, short filaments, and novel oligomer forms [24]. Actin is involved in the export of mRNA transcripts at the nuclear pore complex (NPC) through binding to heterogeneous ribonucleoprotein (hnRNP) complexes [25]. Actin also maintains the stability of the nuclear structure by participating in the regulation of the nuclear lamina and the enhanced adult sensory threshold (EAST) endoskeleton [26]. Additionally, actin is one of the components of the chromatin remodeling complex [13], and actin can change or maintain the chromatin structure [27]. Moreover, super-resolution imaging and RNA-seq reveal that nuclear actin can promote the formation of a transcription factory for inducible genes, which can respond quickly to the external environment [28]. Many reports indicate that actin is present in the nucleus and has a variety of roles in mammalian cells in processes such as transcription, nucleocytoplasmic transport, DNA damage repair, chromatin remodeling, and nuclear structure stabilization [29,30,31,32,33,34,35,36,37,38,39].

Whether plant actin is in the nucleus has been a matter of concern. In fact, a specific form of actin in the nucleus has long been reported [40]. ACT2, ACT7, and ACT8 are distributed throughout the nucleoplasm in isolated nuclei of Arabidopsis, with ACT7 more concentrated in nuclear speckles than the other two [40]. ACT2 and ACT7 aggregate into different types of filamentous structures in Arabidopsis protoplasts and are distributed across the whole protoplast [41]. Moreover, flowering plants have developed a distinctive double-fertilization process accompanied by F-actin-based gamete nuclear migration systems [42]. In Arabidopsis root hairs, the migration direction of cell nuclei is closely linked to the perinuclear actin filaments [43]. On the other side, the actin cytoskeleton plays a crucial role in facilitating intercellular communication through plasmodesmata (PD) [44,45,46]. Supporting this idea, it has been demonstrated that actin and certain actin-associated proteins localize to PD [47,48].

### 2.3. Diverse Functions of Actin Isoforms in Plants

As one of the key components of the cytoskeleton, the monomeric actin plays an irreplaceable role. For example, misexpression of ACT1 leads to dwarfism and altered organ morphology in Arabidopsis. At the highest levels of transgenic overexpression of ACT1, a large number of actin filaments are polymerized, bundled, and reorganized [49], indicating that the abnormal expression of ACT1 in vegetative tissues affects the dynamic arrangement of actin and the orientation of actin-related proteins, disrupting the normal development of plants. Furthermore, misexpression of ACT1 alters the microfilament architecture due to inappropriate interaction of ACT1 with endogenous ABPs [50]. ACT2 is located around the chloroplast and participates in chloroplast photorelocation movement [51]. Similarly, a requirement for ACT7 in actin-dependent chloroplast clustering also has been shown [52].

Actin has been shown to be critical for tip growth in an array of plant models. Plant root hairs achieve tubular shapes through cell tip growth, which requires the coordinated activity of the actin cytoskeleton and endomembrane systems [53,54]. The actin network exhibits rapid turnover near the site of rapid cell expansion. Furthermore, fine actin filament bundles in the apex and subapex are essential for the expansion of the tip, which serve as pathways for the transportation of secretory vesicles to or from the plasma membrane (PM), ultimately aiding in the rapid growth of root hairs. By contrast, thick actin filament bundles in the apical and subapical regions inhibit the growth of root hairs [55]. *ACT2* is a key factor in the correct selection of the bulge site on the epidermal cell and for tip growth of root hair development in Arabidopsis [56]. The *act2-1* mutant generally contains transversely oriented actin filaments, whereas the wild type displays longitudinal actin filaments [57]. Both single-point and T-DNA insertion mutants of *ACT2* affect the length of actin bundles or the polymerization of actin filaments in specific tissues [58,59]. The *act7-1* and *act7-4* mutant alleles show delayed and inefficient germination and roots with increased twisting along with wavy but retarded growth [60]. However, the adult *act2-1*, *act4-1*, and *act7-1* mutant plants are robust, morphologically normal and completely fertile because of functional redundancy [61]. The expression of ACT7 is more relevant to epidermal cell differentiation, cell division, and root architecture, while ACT2 and ACT8 are crucial for regulating root hair tip growth [57]. In the pollen tube tip, the arrangement and dynamic distribution of the actin cytoskeleton are both the powerful motivators of growth and the organizer of cell polarity. *ACT1*, *ACT3*, *ACT4*, and *ACT12* are involved in the dynamic arrangement of actin filaments in germinating pollen grains and tip-growing pollen tubes [62]. Differential regulation of vegetative actin genes and the diversity of actin isovariant sequences are essential for plant development.

The actin cytoskeleton is one of the important factors in plant cell expansion. Thinner and denser arrays of filamentous actin probably participate in expanding cell areas by means of delivering cargo, since the plant actin cytoskeleton is the primary backbone of cytoplasmic streaming [63,64,65]. In Arabidopsis roots, the epidermal and cortical cells have two patterns of rapid elongation: (1) elongating rapidly at the edge of the proximal meristem and transition zone [66,67] and (2) elongating at the boundary between the transition and elongation zones [68]. The second type of rapid cell elongation involves dynamic actin reorganization at the basal end of the transition zone, which is disrupted in the *act7* and *act2act8* mutants [69]. Actually, both the globular actin (G-actin) generated by expression of actin genes and the dynamic structure of the actin cytoskeleton regulated by the ACTIN-RELATED PROTEIN 2/3 (ARP2/3) complex have crucial effects on the second rapid cell elongation [69,70]. In *Gossypium hirsutum*, 15 *GhACT* genes are also differentially expressed in various tissues, among which *GhACT1* is mainly expressed in fiber cells and significantly affects fiber elongation [71]. Additionally, the mutation of the actin gene *Ligon lintless-1 (GhLi1)* disrupts the normal arrangement of the actin cytoskeleton and affects cell elongation, which ultimately leads to formation of various distorted organs [72]. F-actin may act as a trajectory for vesicle movement, thereby regulating fiber cell elongation and secondary cell wall biosynthesis [72]. A general mechanism associated with fine F-actin formation could be involved in accumulating and retaining materials for cell expansion [4]. Another key factor in cell expansion is the auxin signaling pathway, but its association with the actin cytoskeleton remains unclear. During tissue culture, the speed of callus induction in the *act7* mutant is slower under the effect of auxin compared with wild-type callus [73]. A high level of ACT7 protein may be induced by phytohormones to maintain the rapid growth of cell cultures; therefore, ACT7 is necessary for normal callus formation [73]. Additionally, Arabidopsis root meristem development is influenced by ACT7-mediated modulation of auxin-ethylene responses [74]. The unique functional properties of different actin genes may be the evolutionary result of the sessile lifestyle of plants for meeting the demands of various environmental challenges.

**Figure 2:**
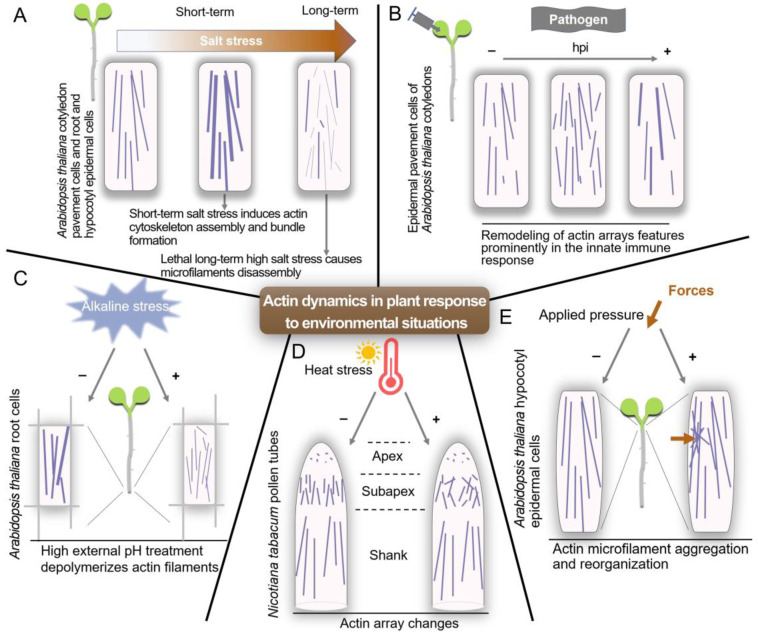
Schematic model of actin dynamics in individual cell growth and plant development in response to environmental stresses. (**A**) Short-term salt stress can trigger the assembly and formation of microfilament bundles in Arabidopsis. However, the polymerization of microfilaments is inhibited in susceptible seedlings subjected to long-term or high salt stress. (**B**) Exposure to *Pseudomonas syringae* pv. *tomato* DC3000 (*Pst* DC3000) induces two distinct changes in the arrangement of the cortical actin network. At 6 h post-inoculation (hpi), cells show an increase in the overall density of actin filament network. Later, ~24 hpi, a reduction in the number of individual filaments or an increase in the extent of actin filament bundling is obvious. (**C**) Under high external pH conditions, microfilament depolymerization is induced, which is associated with the inhibition of Arabidopsis root growth. (**D**) Damages caused by heat stress to actin filaments mainly concern the actin array present in the subapex, a region critical for regulating the organelle and vesicle distribution in the pollen tube apex. (**E**) The Arabidopsis hypocotyl epidermal cell exhibits actin microfilament aggregation in response to mechanical stimulation, and this applied pressure is utilized to simulate the attack by fungal or oomycete hyphae.

Actin also regulates various cellular events in response to external changes. Salt stress-induced microfilament assembly in Arabidopsis is an essential component of salt tolerance [75]. High external pH has no impact on microfilament stability in vitro, but it induces the depolymerization of microfilament in vivo, suggesting that alkaline stress may activate a signal that leads to the reorganization of microfilament [76]. Heat stress affects the organization of actin filaments in the subapex, leading to changes in vesicular transport and cell wall deposition processes [77]. Arabidopsis hypocotyl cells can respond to mechanical stimuli similar to those exerted by fungal and oomycete cells, in which actin microfilaments aggregate and reorganize toward the site of the indentation [78]. Moreover, remodeling of actin arrays features prominently during both early and late events associated with the innate immune response [79]. A summary of the responses of actin filaments under different conditions shows that actin is a key player in enabling plants to adapt and thrive in their ever-changing surroundings (Figure 2).

## 3. Structure and Evolution of the ARP Superfamily

ARPs are named based on their similarity to the canonical actin [10]. The expression patterns of *AtARP5*, *AtARP6*, and *AtARP8* are distinct in seedlings, roots, leaves, flowers, and siliques, whereas expression of *AtARP2* and *AtARP3* was extremely low in all organs [9,11]. The AtARP4 and AtARP7 proteins were concentrated in flowers. Compared with members of the actin protein family, ARPs appear to be more divergent in their predicted structures (Figure 1A). The domains with known functions in the eight AtARP proteins are shown in Figure 1B. The AtARPs were characterized by the nucleotide-binding domain, which are like those mainly present in actin, nucleotide exchange factors, and HSP70 molecular chaperones. The nucleotide sits in a deep cleft generated between the two lobes of the nucleotide-binding domain (NBD), and the residues in the NBD are conserved [80,81,82,83,84,85]. Proteins in the actin and HSP70 superfamilies have functional activities that are regulated by allosteric effectors, which may influence the cleft closure. This conserved domain was prominent in all Arabidopsis ARPs, where the NBD location was a bit different. Overall, the conservation of individual domains in their respective clades may imply their significance for functional specificity.

There is a potential connection between ARP-based chromatin dynamics and the control of diverse developmental processes in plants [86]. *ARP4* may exert its effect on plant architecture, floral senescence, flowering time, and fertility through adjustment of chromatin structure and changes in corresponding gene regulation [86]. *ARP4* and *ARP7* are likely to be involved in chromatin remodeling and transcriptional control during mitotic interphase [87]. ARP4- and ARP5-deficient plants are both hypersensitive to DNA-damaging agents, and a role for ARP5 in DNA repair was demonstrated [88]. ARP5-deficient plants display growth inhibition, morphological changes of individual cells, and abnormal organ development. Presumably, the functions of ARP5 are crucial to normal epigenetic control in Arabidopsis [88]. ARP5 and the chromatin-remodeler INO80 form a larger protein complex that has a synergistic effect in plant cell proliferation and response to replication stress [89]. At the same time, *INO80* and *ARP6* collaborate in embryogenesis and postembryonic plant development, while the synergy of *ARP5* and *ARP6* contributes to the maintenance of genomic stability [89]. Likewise, the *arp7* mutant and *ARP7* RNAi lines show abnormalities at different stages of plant growth and development due to the epigenetic impact of ARP7 complexes on chromatin-mediated regulation of gene expression [90,91].

Functional investigations of ARP proteins show that they form stable hetero-multimeric protein complexes with other proteins [92]. For instance, ARP2/3 complexes are implicated in generating the actin arrays related to polarized growth [11,93]. In leaf pavement cells of Arabidopsis, the ARP2/3 complex covers the surface of organelles that can efficiently bind to actin filaments and the microtubule cytoskeleton, meaning that the function of ARP2/3 affects the structure of G- and F-actin during reconfigurations of the cytoskeleton [94]. ARP2/3 is intrinsically inactive [95], but its complex can be converted to a potent actin filament nucleator via interaction with nucleation promoting proteins, such as the Wiskott-Aldrich syndrome protein (WASP)/Scar homolog (WASH), the WASP homolog associated with actin, membranes and microtubules (WHAMM), and the junction-mediating regulator protein (JMY) [96]. ARP3/DISTORTED1 (DIS1) acts in amyloplast sedimentation through altering apparent local viscosity in the central columella cells and redistributes asymmetric auxin in the root tip by modification of PIN-FORMED (PIN) protein trafficking [97]. In plants, formin homologs and the ARP2/3 complex are the known actin nucleators. These two actin nucleation systems, the *Arabidopsis thaliana* FORMIN HOMOLOGY 1 (FH1) and the ARP2/3 complex subunit 5 (ARPC5), have complementary or parallel functions in terms of some aspects of cell morphogenesis [98]. The ARP2/3 complex, together with FH1, mediates actin patch formation, thereby contributing to host cellular defenses and penetration resistance against fungal invasion [99]. The ARP2/3 complex precisely regulates guard cell actin remodeling and stomatal movement in order to ensure an appropriate stomatal aperture in response to environmental challenges [100]. Additionally, the ARP2/3 complex can participate in mitochondrial-associated calcium signaling pathway to respond to salt stress [101]. In brief, most of our understanding of distinct ARP functions is based on how various ARP-containing complexes work, but our knowledge of their functional characteristics in plants is still in its infancy. It would be interesting to explore whether there are differences between animal and plant ARP features.

## 4. Role of ABPs: An Accurate Network Controller of Plant Actin Dynamics

Under normal conditions, about 95% of the actin cytoskeleton is composed of monomeric actin in a state of readiness [102,103]. The actin monomer is a core part of the microfilament skeleton and can receive signals or be regulated by actin monomer binding proteins, so that the actin cytoskeleton can quickly and accurately respond to changes in the external environment. Concurrent with the evolution of actin, certain specific ABPs also gradually developed [104]. Different ABPs not only regulate actin activity, but also play an important role in promoting nucleation, which controls the assembly and formation of microfilaments (Table 1). ABPs are divided into two categories according to their function: one category keeps the balance of an actin monomer pool through affecting the dynamics and assembly of the unit structure of microfilament; the other category can arrange the microfilament into more complex structures [105]. Here, we highlight the main classes of ABP found in plant cells and suggest their likely mechanism of action, as far as possible, based on both in vitro or in vivo studies.

### 4.1. Fimbrin

Fimbrins possess the actin-binding domain (ABD) composed of two tandem calponin-homology (CH) domains. Each fimbrin contains two ABDs, enabling it to crosslink actin filaments as a monomer and generate high-order actin structures [128,129]. Arabidopsis has five FIMBRIN genes, with FIMBRIN1 (FIM1) and FIM5 being crucial for maintaining the polarity of actin bundles in pollen tubes and playing a role in pollen development [120,121,130]. FIM4 acts in coordination with FIM5 to organize and maintain normal actin architecture in pollen tubes and pollen grains, thus fulfilling double fertilization in Arabidopsis [131]. Additionally, fimbrin-dependent cross-linking plays a significant role in creating robust microfilament bundles that facilitate cytoplasmic streaming. Fimbrin serves to safeguard these bundles from severing and depolymerizing agents [121,132]. Due to the distinct tissue expression patterns and biochemical activities observed in Arabidopsis fimbrins, it is believed that they function in fulfilling diverse actin-based physiological cellular processes [133,134].

### 4.2. Formin

Formin is a type of actin nucleation factor that has been implicated in the formation of linear actin bundles [135]. Formin proteins are distinguished by the presence of FH1 and FH2 domains, which have the ability to nucleate actin assembly from actin or actin-profilin complexes [135]. The formins have been implicated in many actin-based cellular processes in plants, including root growth, polarized pollen tube growth, cell division, cytokinesis, cell morphogenesis, and plant defense [136,137,138,139,140,141]. In Arabidopsis, there are a total of 11 class I formins and 10 class II formins. Among these, the class I formins possess a distinctive transmembrane domain at their N-terminus, allowing them to specifically target membranes [142]. During the ontogeny of root cells, FH1 undergoes relocation between membrane compartments and forms associations with PD [143]. FH2 regulates PD permeability by anchoring actin filaments to PD, which is crucial for normal intercellular trafficking [144]. In addition, rice formin protein OsFH13 is a putative traffic protein from the PM to the chloroplast membrane and bridges the actin cytoskeleton and light signaling [145]. A central mechanism in plant immune signaling suggests that rapid actin remodeling occurs through the nanoclustering of formin integrated into the PM [146,147]. In short, formins might have a shared role at cell–cell junctions in plant.

### 4.3. Capping Protein

Highly conserved homologs of capping protein (CP) are present in almost all eukaryotic cells, including higher plants, fungi, and various cells and tissues in vertebrates [127]. In vitro, CP, as a heterodimeric protein complex, binds tightly to the barbed ends of actin filaments, effectively preventing the addition or loss of actin subunits [127]. As actin polymerization occurs, AtCP is an efficient nucleator of actin filament formation from monomers and shortens the delay period prior to actin assembly. The activity of AtCP is not affected by calcium, but it does exhibit moderate sensitivity to the signaling lipid phosphatidylinositol 4,5-bisphosphate [PtdIns(4,5)P_2_] [127]. In fact, CP is a phosphatidic acid biosensor and converter of fluxes of membrane-signaling phospholipids into dynamic changes in the actin cytoskeleton [148]. During plant innate immunity, the negative regulation of CP by phosphatidic acid plays a vital role in actin remodeling, and CP also act as an intermediary in ROS signaling to the actin cytoskeleton [149,150]. Moreover, AtC α and β subunits (i.e., *AtCPA* and *AtCPB*) show distinct expression patterns in vivo, and the downregulation of *AtCPB* leads to enhance thermotolerance in plants following exposure to heat shock stress [151].

### 4.4. Villin

Villins regulate actin by promoting actin-bundling [152]. These actin-bundling proteins crosslink adjacent actin to bundle several parallel actin filaments [153]. In Arabidopsis, there are five VILLINs divided into three functionally distinct groups [154]. Even when plant cells receive signals that lead to the depolymerization of actin arrays, such as actin-depolymerizing factor (ADF)-mediated actin filament depolymerization, VILLIN1 maintains the cable network by regulating actin filament bundle formation and stability [115]. Nevertheless, ADF7 inhibits VILLIN1 to modulate F-actin dynamics in root hair formation in response to osmotic stress [155]. VILLIN3 phosphorylation by MITOGEN-ACTIVATED PROTEIN KINASE 3/6 (MPK3/6) regulates actin remodeling to trigger stomatal defense in Arabidopsis [156]. Besides bundling F-actin, VILLIN3 can sever actin filaments in a Ca^2+^-dependent manner and promote bundle turnover [154]. Similarly, the regulation of long axial and short apical actin bundles by VILLIN4 is important for cell growth and cytoplasmic streaming in root hairs, which is also influenced by Ca^2+^ signaling [6]. VILLIN4 plays a crucial role in regulating the dynamics of PIN2 in response to 2,3,5-triiodobenzoic acid, supporting the important role of actin dynamics in the mechanism of auxin transport [157]. In addition, the unifying mechanism behind the VILLIN2- and VILLIN5-mediated regulation of actin dynamics in the apical dome may be that VILLIN proteins sever the actin filaments at the apex of pollen tubes in conjunction with an apically focused Ca^2+^ gradient [114,158]. These actin bundles are essential for cell expansion during directional organ growth [159]. Given that VILLINs perform multiple actions as Ca^2+^-responsive and F-actin regulatory proteins, it will be challenging to investigate how Ca^2+^ signaling affects the actin dynamics through the integration of VILLINs activity.

### 4.5. LIM Domain-Containing Protein

LIM domain-containing proteins (LIMs), a class of LIM domain proteins related to animal Cys-rich proteins, have been reported to initiate the formation of actin bundles, a significant assembly of the higher-order cytoskeleton [125,126]. In the six *Arabidopsis thaliana LIMs*, *WLIM1*, *WLIM2a*, and *WLIM2b* are widely expressed, whereas *PLIM2a*, *PLIM2b*, and *PLIM2c* are predominantly expressed in pollen [160]. Furthermore, the balance between PLIM2a/PLIM2b and FIMBRIN5 (FIM5) is essential to maintain the proper organization and normal bundling of longitudinal actin bundles in pollen tubes [122]. LIMs contribute to the regulation of actin bundling in virtually all plant cells as a highly specialized ABP family [160]. In lily pollen tubes, LlLIM1 plays a rational role in integrating endomembrane trafficking with a pH- and calcium-sensitive manner [125].

### 4.6. Myosin

The actin cytoskeleton and relevant motor proteins underlie the movement of plant organelles and multiple materials within plant cells. Two myosin families, VIII and XI, drive cytoplasmic streaming, organize actin, and regulate cell expansion [161]. Myosin VIII (four genes in *A. thaliana*) in the roots of maize and cress localizes on the PD and at newly formed cell plates [162]. Myosin VIII may participate in intercellular transport through PD [163]. Enzymatically, myosin VIII can generate or sense tension, and this tension makes the PD better for cargo transference and junction formation between the endoplasmic reticulum and the PM [45,164]. Among the 13 *A. thaliana* myosin XI isoforms, myosin XI1, XI2, and XIK generate force that enables the buckling and straightening of actin filaments and bundles, as well as facilitate actin filament turnover [165]. Moreover, myosin XI functions in vesicle exocytosis and cellulose production at the cytoskeleton-PM-cell wall nexus [166]. Specifically, myosin XIK associates with secretory vesicles earlier than the exocyst, and it is likely responsible for recruiting or stabilizing the exocyst at the PM tethering site to facilitate vesicle tethering [167]. Myosin XIG regulates the meshwork F-actin movement via myosin functions that are distinct from organelle movement [42]. The four functional domains of myosins enable them to glide along actin filaments using energy from ATP hydrolysis and then to transport organelles or protein complexes. It is the close relationship between myosins and cytoplasmic streaming that plays an important role in the polar growth by affecting directional tip elongation of typically long cells, such as root hairs, pollen tubes, and moss protonemata [168,169,170]. Moreover, myosin function in cell division is distinct from driving cytoplasmic streaming [171]. Because organelle traffic and cytoplasmic flow occur in both growing and fully differentiated cells, the functional allocation between the molecular motor- and the membrane receptor-dependent pathway needs to be further confirmed while these processes facilitate intracellular homeostasis [3].

### 4.7. Profilin

Profilin (PRF) has a profound influence on the organization of the actin cytoskeleton mainly through sequestering actin monomers to maintain the level of polymerizable actin monomers. PRF can bind to proteins containing proline-rich sequences and phospholipids, which further drives PRF to bind actin monomers in multiple ways. The conserved domain in PRFs is the PRF-actin interacting region (PAINR), which plays vital roles in the binding process [172,173]. PRFs, in direct or indirect interaction with membranes, transmit information between the actin cytoskeleton and the PM via PHOSPHATIDYLINOSITOL 4, 5-BISPHOSPHATE (PIP_2_) [118,174]. PRFs can affect fiber growth in cotton, cell shape maintenance in Arabidopsis, as well as flowering time in tobacco [175]. In addition to influencing various aspects of cellular development, PRFs also positively contribute to management of stresses, such as salinity stress [175,176]. PRFs may coordinate with formins during actin polymerization. The polyproline tract sequences located in FH1 enable the profilin-actin complex to bind FH1 [177,178]. During plant cell expansion, PRF1 coordinates the stochastic dynamic of actin filaments by modulating formin-mediated actin nucleation and assembly [179]. AtPRF3 is an atypical isoform of profilin found in Arabidopsis, and it possesses an N-terminal extension that results in protein oligomerization and hampers the formin-mediated actin assembly [180]. Furthermore, AtPRF3 regulates the immune responses triggered by pathogen associated molecular patterns (PAMPs), which in turn also influence the degradation of AtPRF3 [180]. In pollen germination, PRF4 and PRF5 control vesicle movement and polarity establishment by facilitating FH5-mediated actin polymerization and strengthening the interaction between FH5 and actin filaments [181].

### 4.8. Cyclase-Associated Protein

Another class of proteins that can bind actin monomers is the cyclase-associated proteins (CAPs), which perform multiple functions due to their two distinct domains that bind adenylyl cyclase and G-actin at the N- or C-terminus, respectively [182]. In contrast to plant PRFs, CAP1 is capable of directly accelerating the nucleotide exchange on G-actin, even without the role of ADF/cofilin [183]. It has been speculated that coupling between ADF/cofilin-mediated depolymerization of actin filaments and PRF-mediated assembly of ATP-G-actin needs a CAP protein as a key intermediate [123]. CAP is also involved in the process of regulating cell expansion by the actin cytoskeleton in Arabidopsis. Both the number and size of leaf cells are altered through overexpression of *CAP*, which causes a reduction in organ size [184]. During normal pollen tube growth, CAP1 is an abundant cellular protein that acts in concert with PRF and ADF to enhance actin turnover and ADP-G-actin nucleotide exchange in vitro. The change of apical actin polymerization has differential effects on various regions of pollen tubes by altering the actin cytoskeleton [185]. In addition, a defect of CAP1 alters the developmental tendency of multiple cell types, such as meandering roots and curling inflorescences [186].

### 4.9. Actin-Depolymerizing Factor

The ADF family contains well-characterized ABPs that can change orientation and arrangement of actin filaments through binding monomeric actin or modifying filamentous actin [187,188]. The diverse tissue expression patterns of the 11 Arabidopsis *ADFs* suggest that they have evolved different physiological characteristics [189].

In pollen tubes of Arabidopsis, ADF7 evolved to promote turnover of longitudinal actin cables through severing actin filaments that might occur in the actin fringe [190,191]. ADF10 arranges filamentous actin around the pore of the mature pollen grain in the developing gametophyte [192] and promotes the circulation and arrangement of the apical actin filament to regulate vesicle trafficking and pollen tube growth [193]. In *Nicotiana tabacum*, overexpression of *NtADF1* in elongating pollen tubes disrupts the actin cytoskeleton and causes a decrease in growth rate [194]. In *Physcomitrium patens*, F-actin organization is altered with loss of ADF function, resulting in an inhibition of tip growth [195]. Phospho-regulation at serine 6 is a requisite for effects of ADF on polarized growth [195]. Beyond that, an altered expression level of *ADF1* or *ADF9* affects F-actin organization, flowering time, and cell expansion in Arabidopsis [196]. ADF9 is an actin-bundling protein whose activity is adjusted by pH conditions and antagonizes ADF1 activity through reducing its ability to potentiate F-actin depolymerization [110]. ADF9 is a novel photoperiod-dependent early flowering repressor, which is regulated by *CONSTANS* (*CO*)- and *FLOWERING LOCUS C* (*FLC*)-related networks [196,197]. ADF4 plays a role in the process of modulating actin filament turnover, and the *adf4* mutant displays altered cytoskeletal arrays and morphologies of hypocotyl and epidermal cells [198].

The functions of ADF family proteins are implicated in response of plants to environmental stimuli, but the evidence is indirect or limited. ADF4 has been identified as a signaling component that can transport the *Pseudomonas syringae* effector proteins Avirulence protein *Pseudomonas phaseolicola* B (AvrPphB), and RESISTANT TO PSEUDOMONAS SYRINGAE 5 (RPS5) around the PM by mediating rearrangement of the actin cytoskeleton, which aids in the subsequent identification of cargo [199]. Inhibition of ADF4 activity during innate immune signaling can modulate actin dynamics so as to implement PAMP-triggered immunity (PTI)-related response strategies in Arabidopsis [200]. ADF4 is a physiological substrate of CALCIUM-DEPENDENT PROTEIN KINASE 3 (CPK3), and phosphorylation of ADF4 by CPK3 controls actin cytoskeletal organization associated with pattern-triggered immunity [201]. During the response to abiotic stress, ADF5 may be involved in the ABA signaling pathway and regulate the actin cytoskeleton to affect stomatal movement in response to drought stress [202]. Under a low-temperature environment, C-REPEAT/DRE BINDING FACTORs (CBFs) can bind to the CRT/DRE DNA regulatory element of the ADF5 promoter, which activates the expression of *ADF5* and modulates the actin cytoskeleton dynamics [203]. The opposing and diverse biochemical properties of plant ADFs are caused by evolutionary changes in key amino acids [106]. The regulatory mechanism of ADF activity in the organization of F-actin and the correlation between ADF expression and nuclear function should be investigated in more detail.

## 5. Signals and Pathways Regulating the Actin Cytoskeleton

**Figure 3:**
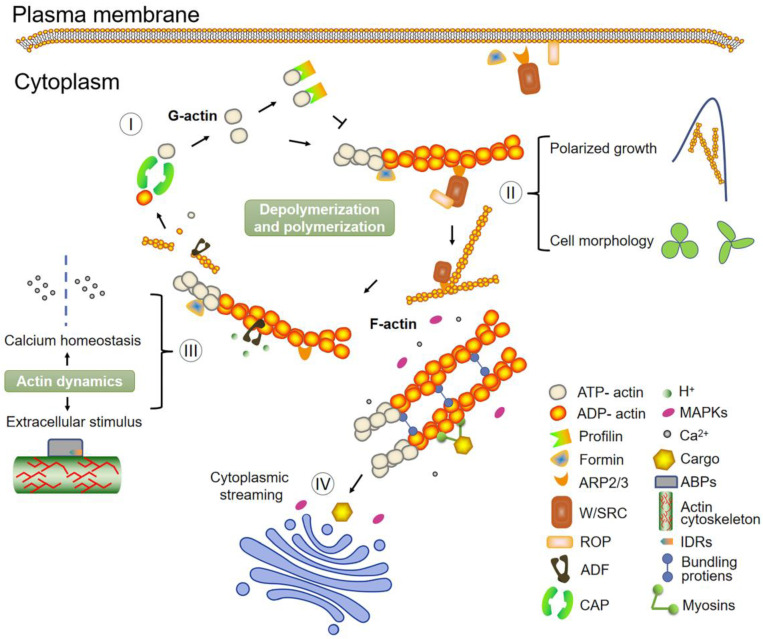
Fundamental model depicting actin dynamics and various processes that involve the actin filaments. (I) The conversion from ADP-actin to ATP-actin is accomplished by CAP and is a strategic step that guarantees the polymerization cycle. The nucleation factors, like ARP2/3 and the formins, order the polymerization of the actin monomers (G-actin). Profilin can also bind actin monomers to maintain the actin monomer pool by inhibiting the polymerization of actin filaments. Generated actin filaments (F-actin) are bundled at different angles via binding proteins or crosslinkers. Any depolymerized actin filaments will re-enter the assembly system. (II–IV) The dynamic changes of the actin cytoskeleton participate in plant cell development through the functional association of different signaling pathways. Filamentous actin arrays are associated with plant cell growth, and the activity of ABPs is essential for proper cell morphogenesis. Cytoplasmic streaming plays a vital role in the transportation of materials necessary for tip elongation. Meanwhile, actin reorganization during cellular processes, such as polar growth and cytoplasmic streaming, is frequently correlated with calcium influxes or intracellular calcium gradients.

Many direct correlations between the actin cytoskeleton reorganization and signal transduction exist in plants, including polarized growth, Ca^2+^ homeostasis, cytoplasmic streaming, and responses to extracellular stimulus (Figure 3). A focus of research in this area will make it easier to analyze the evolutionary direction of actin function.

The RHO-RELATED GTPases (ROPs) is a family of small signaling GTPases in plants that can transmit specific signals to the actin cytoskeleton in response to intracellular and extracellular signals [204,205]. Fine actin architecture and ROPs are localized in the tip area of apical cells. Multidimensional cell expansion during early stages of tissue development may be regulated by a related mechanism involving ROP signaling-dependent formation of cortical F-actin [206,207]. Downstream of the ROP and phosphoinositide (PI) signaling pathway, Ca^2+^ concentration and pH level modulate ABPs, which interact with phospholipids and several critical proteins in a phosphorylation-dependent manner [208,209,210]. Moreover, activity of ARP2/3 is positively regulated by the WASP FAMILY VERPROLIN HOMOLOGOUS PROTEIN (WAVE) complex that may be a ROP2 effector complex [211]. The readjustment of actin filament dynamics under the WAVE-ARP2/3 pathway can guide the polar growth of early stage trichomes [211]. These two complexes function in nucleating actin filaments, which ultimately leads to changes in cell morphology.

Reactive oxygen species (ROS) not only serve as significant stressors for cellular components but also play diverse roles in cell physiology [212]. There are the crucial connections between ROS signaling and actin cytoskeleton [213]. ARP2/3-mediated actin dynamics are essential for stomatal movement in response to ABA-induced ROS signaling [100,214,215]. Actin arrays in guard cells of *respiratory burst oxidase homologues D/F* (*rbohD/F*) mutants treated with ABA fail to reorganize, whereas applying H_2_O_2_ to *rbohD/F* mutants recapitulates actin remodeling in the absence of ABA [215]. PHOSPHOLIPASE Dα1 (PLDα1)-generated phosphatidic acid (PA) functions upstream and binds directly to RBOHD, resulting in the production of ROS during ABA signaling and subsequent stomatal closure [216]. In addition, exogenous ROS treatment induces the accumulation of actin filaments in leaf epidermal cells. During plant innate immunity, it has been found that CP is essential for transducing RBOHD/ROS signaling to facilitate actin remodeling in response to flg22, a 22-amino-acid epitope derived from flagellin [150]. Since PA regulates actin remodeling through CP, there is a negative feedback regulation by CP or the actin cytoskeleton to modulate ROS production elicited by flg22 [217].

The plant hormone cytokinin (CK) plays pivotal roles in plant development and throughout plant life. Root system architecture induced by CK involves the reorganization of actin filament [218]. In particularly, the transition from the transition zone (TZ) to the elongation/differentiation zone (EDZ) is an important mode of rapid cell elongation in epidermal and cortical cells of Arabidopsis roots [69]. During this time, CK promotes actin bundling and the resultant cell elongation through activating the ARABIDOPSIS HISTIDINE KINASE 3/4 (AHK3/4)-ARABIDOPSIS RESPONSE REGULATOR 2 (ARR2) pathway [69]. Additionally, the impact of CK on the actin cytoskeleton could lead to changes in trafficking rates and paths for endomembrane compartments, which could affect the distribution of defense-related cargo and result in altered defense signaling [219]. Thus, the modulation of the cellular cytoskeleton and trafficking could potentially serve as a mechanism that executes downstream responses of CK signaling [219].

The plant chloroplast and nucleus change position in response to light. Plant cells have seemingly evolved distinct mechanisms to regulate actin organization, which is necessary for driving the movements of these organelles [220]. In Arabidopsis leaf cells, blue-light-dependent nuclear positioning is regulated by the blue light receptor PHOTOTROPIN 2 (PHOT2)-dependent reorganization of the actin cytoskeleton [221]. The chloroplast-actin filaments (cp-actin filaments) emerge from the chloroplast edge and display rapid turnover. When chloroplast movement is induced by blue light, the cp-actin filaments undergo relocalization to the leading edge of chloroplasts both before and during photorelocation and are regulated by PHOT1 and PHOT2 [222]. THRUMIN1 plays a key role in connecting phototropin photoreceptor activity at the PM with actin-dependent chloroplast movements [223,224]. Moreover, the spatial reorganization of F-actin is also affected by red light in water plants, where chloroplast movements are closely linked with cytoplasmic streaming [225]. In *Physcomitrella patens*, KINESIN-LIKE PROTEIN 1/2 (KAC1/2) mediate the actin-dependent chloroplast light avoidance response [226]. Therefore, actin-based mechanism is important for light signaling-directed organelles movement.

MITOGEN-ACTIVATED PROTEIN KINASEs (MAPKs) signal transduction is involved in nearly all regulation of crucial cellular processes. The cross-talk between actin dynamics and MAPK signaling exists in response to environment stimuli and cell shape and morphogenesis in plants. In alfalfa, STRESS-INDUCED MAPK (SIMK) and STRESS-ACTIVATED MAP KINASE (SAMK) are involved in responses to osmotic, cold, and heat stress. They are activated when the actin cytoskeleton is disrupted through treatment with microfilament depolymerising drugs [227,228]. MAPK signaling pathways may be the key sensor for balancing the intracellular forces used for controlling cellular architecture [229]. In *Papaver rhoeas*, self-fertilization and consequent inbreeding rely on the specific self-recognition of pollen regulated by self-incompatibility (SI) pathways. SI-induced tip growth inhibition may be accomplished through an alteration of F-actin by a Ca^2+^-dependent signaling cascade [230,231]. In addition, Ca^2+^ can modulate ABP activity by directly binding them or indirectly through Ca^2+^-stimulated protein kinases, such as calcium-dependent protein kinases (CDPKs) [208,209]. On the other hand, actin dynamics can regulate the activity of Ca^2+^-permeable channels to maintain Ca^2+^ homeostasis [232].

**Figure 4:**
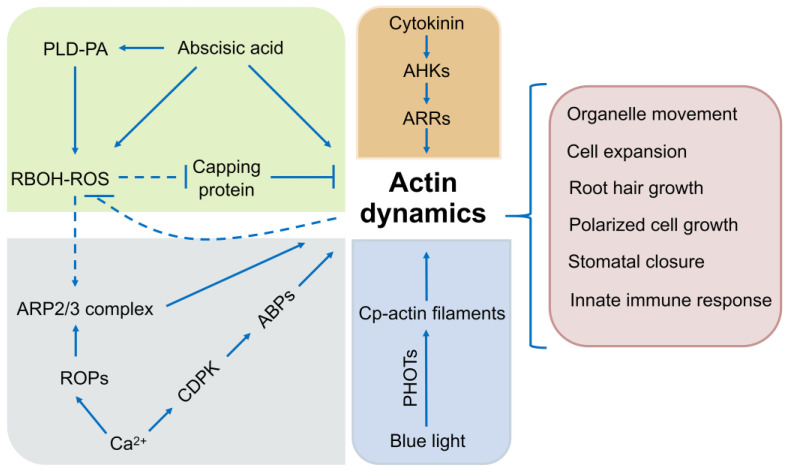
Potential connections between the actin cytoskeleton and signaling molecules, involving ROS signaling, Ca^2+^ signaling, light signaling, cytokinin signaling, and ROP signaling. This scheme is based on published research data discussed in this review. Solid lines, direct interaction; dashed line, potential interaction; arrows, activation of signaling cascades; bars, inhibitory effect; PLD, PHOSPHOLIPASE D; PA, phosphatidic acid; RBOH, RESPIRATORY BURST OXIDASE HOMOLOGUE; CP, chloroplast; AHK, ARABIDOPSIS HISTIDINE KINASE; ARR, ARABIDOPSIS RESPONSE REGULATOR; ARP2/3, ACTIN-RELATED PROTEIN 2/3; ROP, RHO-RELATED GTPase; CDPK, calcium-dependent protein kinase; ABP, actin-binding protein.

Intrinsically disordered regions (IDRs) are present in a majority of ABPs and affect the activity of ABPs indirectly by altering conformational changes during complex protein assembly [233]. In a sense, IDRs can create diverse and flexible interactions between ABPs and the actin cytoskeleton to influence various signal transduction events [233,234,235]. The distinctive characteristics of IDR-mediated plant actin remodeling would enrich our comprehension of the structure–function relationships involved in actin assembly [236]. In summary, the actin cytoskeleton can be thought of as a ubiquitous downstream signal effector that participates in plant individual growth and system development (Figure 4). The further understanding of key links between such signaling cascades and actin dynamics is a top priority.

## 6. Support for In-Depth Investigation on Plant Actin

Actin is one of the most highly conserved proteins across multiple kingdoms of life, with an 80% sequence conservation at the gene level between humans and the yeast *Saccharomyces cerevisiae* [104]. In mammalian cells, nuclear actin can regulate the transcription of induced genes by enhancing the aggregation of RNA polymerase II [28]. Actin genes in plant species have been identified by in silico analysis and other research approaches [237,238]. Whether it is the application of 3D culture systems based on organoids or the extension of super-resolution microscopy through fluorescence observation [239], animal cell materials seem to be more convenient for use of these new technologies and methods compared with plant cells. The further development of cryo-electron microscopy has made it possible to more accurately explore the structure of filamentous actin [240]. Using *Zea mays* pollen and rabbit skeletal muscle actin filaments, single-molecule magnetic tweezer and structural data analyses have found that plant actin filaments are more stable compared with animal actin filaments [241]. Differences in structure may determine the many functional differences between mammalian and plant actins that arose during their evolution. Therefore, it is a long process to further understand the dynamic regulation of actin in multiple plant cellular functions.

The cytoskeleton is an influential factor in the dynamics of subcellular membranes, organelle movement, membrane trafficking, and cellular morphogenesis [242,243,244]. However, not all proteins identified in animal systems to control membrane dynamics and the cytoskeleton operate similarly in plants, especially those proteins that act as membrane-actin adaptors. The mechanism of action of the actin-binding proteins in the NETWORKED (NET) superfamily represents an important field for evaluating plant actin cytoskeleton-endomembrane interactions [245]. In addition, different stages of autophagosome formation require a functional actin network as a support for vesicle trafficking and membrane fusion, which has been demonstrated in animal and yeast cells [246,247,248]. The function of the ARP2/3 complex in plant and mammalian cells is overall different [124,249]. In plants, the ARP2/3 complex and the related activators, the SCAR/WAVE and AtEH/Pan1 complexes, can inhibit autophagosome biogenesis when mutated, resulting in decreased abiotic stress resistance [250,251,252]. Although some autophagosome-actin associations have been summarized into detailed models [253], it remains to be confirmed whether the function of actin in autophagosome formation is independent or irreplaceable. Indeed, various mechanisms of action involving the actin cytoskeleton are conserved among organisms, but there are some notable exceptions, both in signaling components and in physiologically relevant links. Many actin-binding/regulatory proteins involved in apoptotic signaling pathways in mammalian cells have not been identified in yeast and do not exist in the Arabidopsis genome database [254]. Admittedly, it may be of considerable interest to investigate whether proteins with similar sequences or activities also perform similar functions across species.

The ability to fluorescently label proteins has allowed the increasingly accurate examination of the dynamic distribution of the actin cytoskeleton in living plants [15,255]. Meanwhile, advanced fluorescence microscopy has provided super-resolution approaches that have improved the spatial and temporal resolution of plant cell dynamics [256,257]. A combination of high-speed F-actin co-sedimentation assay and total internal reflection fluorescence microscopy (TIRFM) imaging technology effectively achieves direct visual analysis of actin filament severing [258]. For example, ACTIN-INTERACTING PROTEIN 1 (AIP1) and ADF synergistically regulate the turnover of actin filaments within the growth zone of pollen tubes, which pushes forward construction of the unique apical actin structure at the pollen tube tip [258]. Additionally, to comprehensively understand the cellular functions of actin organization and dynamics, some parameters have been created to quantify the organization of actin filaments, such as slope, filament density, and skewness [259,260]. From this perspective, the technological and inquisitive advances hold great promise for grasping the intricate details related to the actin cytoskeleton regulatory network at a molecular scale.

Action potentials are fundamental for facilitating long-distance signaling in plants and animals, and their functional linkage with the actin cytoskeleton associated with membranes is of utmost importance [261]. The actin cytoskeleton functions downstream of action potentials, which are accomplished through excitable membranes. Action potentials directly affect the lipid bilayer phospholipids and the proteins embedded within the PM. These include ion channels and transporters, as well as transmembrane proteins that directly regulate actin polymerization, such as formins [136,262,263,264,265]. In the root apex transition zone, the cells with the highest rates of electric spikes are the ones that assemble dense F-actin meshworks through formin activities. These meshworks play a critical role in supporting endocytosis and the recycling of endocytic vesicles [262,264,265]. Besides formins, myosin VIII may be relevant for the propagation of the action potentials [266,267]. The plant-specific actin-based endocytic motor is involved in the myosin-actin-based gating of plant plasmodesmata, which is important for the transmission of action potentials between cells [268,269,270]. Action potentials regulate various aspects of plant life [261]. Precise connections between the inherent bioelectricity of eukaryotic membranes and the actin cytoskeleton are still under-investigated, and studies in this field serve as a fundamental pillar in our pursuit to comprehend actin-related cellular life activities.

## 7. The Coordinating Effect of Microtubules and Microfilaments

Microtubules were originally viewed as a network with separate functions distinct from actin filaments. However, microfilaments and microtubules have repeatedly been found to work together in yeast and animal cells [271]. The dynamic interaction between actin filaments and microtubules in Arabidopsis has been confirmed by ultra-clear images derived from confocal microscopy [272,273], suggesting that there must exist indirect or direct connections. ABPs and microtubule-associated proteins (MAPs) are major players determining the spatiotemporal dynamics of microfilaments and microtubules and act as sensory hinges that converge different signals to regulate plant cytoskeletal behavior. For instance, the actin binding protein NETWORKED 3C (NET3C) interacts with two microtubule binding proteins, IQ67-DOMAIN 2 (IQD2) and KINESIN LIGHT CHAIN-RELATED PROTEIN 1 (KLCR1), to form a novel module for the organization and maintenance of endoplasmic reticulum (ER) morphology and cytoskeletal structure [274]. Members of the MICROTUBULE DESTABILIZING PROTEIN (MDP) family, MDP25 and MDP18, are mediated by Ca^2+^ signaling and affect microfilaments and microtubules during pollen tube growth [5,275,276,277]. MDP25-mediated actin dynamics are associated with MDP25 function under salt stress [278]. In addition, phragmoplasts are responsible for plant cytokinesis during cell expansion, and this structure is composed of microtubules, actin filaments, and membrane vesicles, all of which support the formation and expansion of the cell plate [279,280]. Cell plate formation is delayed by the breakdown of actin filaments, a process that may be linked to interruption of the initial phragmoplast microtubules [281]. In terms of polar growth, microtubules can direct the localization of actin nucleation factors, and the resulting actin filaments further focus the microtubules [282]. This process creates a positive feedback loop that brings actin polymerization and cell expansion together at a proper location to support sustainable polar growth [282].

## 8. Conclusions and Future Outlines

This review provides a wide discussion in regard to how actins, ARPs, and ABPs work together to drive cellular functions. Understanding of the multiple ways that F-actin influences the regulation of various cellular functions is one of the ultimate research goals in this field. The first further inquiry is finding out what higher-order structures are established by actin monomers and how the dynamic balance of the entire actin filament is maintained. Then, the numerous ABPs, including novel ABPs that have not yet been discovered, should be studied, including how microfilaments function and integrate into complex signal transduction processes. Because the components related to the actin cytoskeleton are highly conserved across many species, we can deduce functions through the subtle differences between sequences and work out similarities and connections to cellular components. Meanwhile, the development of super-resolution technologies and experimental strategies can be used to reveal the specific roles of the actin cytoskeleton in vital activities.

In plants, the signaling between actin and microtubules occurs all the time for many cellular processes. Many specific proteins ensure that the pivotal mechanisms that regulate microtubule–actin interactions operate. The proteins are conserved across diverse eukaryotes, which can serve as a major direction for follow-up investigations. It is also of interest to figure out what coordination between regulatory proteins modulate actin and microtubule dynamics under physiological conditions and how plants respond to extracellular challenges by harmonizing the activities and levels of ABPs and MAPs. These questions seem to point to a number of mechanisms underlying the control of MAPs and ABPs in cytoskeletal organization and dynamics. Finally, the newest 3D in vivo imaging combined with diverse fluorescent biosensors can be used to explore the above problems [273,283,284,285] and to address the gaps in our understanding of functional microtubule–actin interactions. Further dissection of cytoskeleton-related signaling mechanisms should enable plant improvement, supporting the development of sustainable and enhanced crops.

## Figures and Tables

**Table 1:** Biochemical properties of representative plant ABPs in actin cytoskeleton.

ABP Types	Species	Proteins	Activities or Effects on Actin Cytoskeleton	References
Actin-depolymerizing factor	*Arabidopsis thaliana*	ADF1, 2, 3, 4, 6, 7, 8, 10, 11	Severing or depolymerizing actin filaments	[106]
	*Zea mays*	ADF3	Severing or depolymerizing actin filaments	[106]
	*Zea mays*	ADF1, ADF2	Involving in pollen actin reorganization	[107,108]
	*Lilium longiflorum*	ADF1	Severing or depolymerizing actin filaments	[109]
	*Arabidopsis thaliana*	ADF9, ADF5	Actin-bundling and actin-stabilizing activities	[110]
	*Nicotiana tabacum*	ADF1	Actin-binding ability	[111]
Villin	*Lilium longiflorum*	P-115-ABP, P-135-ABP	Actin-bundling activity	[112]
	*Arabidopsis thaliana*	VLN2, VLN3, VLN4	Being responsible for actin bundle formation	[113]
	*Arabidopsis thaliana*	VLN5	Harbor filament bundling, barbed-end capping, and Ca^2+^-dependent severing activities	[114]
	*Arabidopsis thaliana*	VLN1	Generating actin bundles and stabilize actin cables	[115]
Formin	*Arabidopsis thaliana*	FH1	Inducing supernumerary actin cable formation	[116]
	*Arabidopsis thaliana*	FHs	Nucleating, bundling and severing actin filaments	[117]
Profilin	*Arabidopsis thaliana*	PRF1, PRF2	Having high affinities for both PLP and G-actin	[118]
Myosin	*Arabidopsis thaliana*	MYOSIN XI-K, XI-1, XI-2	Cargo, actin-binding, and ATPase activities; Providing the tensile force to pull an actin filament straight	[119]
Fimbrin	*Lilium longiflorum*	FIM1	Stabilizing the actin fringe by cross-linking actin filaments into bundles	[120]
	*Arabidopsis thaliana*	FIM1	Organizing actin filaments into loose networks	[121]
	*Arabidopsis thaliana*	FIM5	Organizing actin filaments into tight actin bundles	[122]
Cyclase-associated protein	*Arabidopsis thaliana*	CAP1	Nucleotide exchange activity	[123]
Actin nucleation factor	*Arabidopsis thaliana*	Arp2/3 Complex	Enhancing actin nucleation and polymerization and initiating the formation of a dynamic, dendritic array of F-actin	[124]
LIM domain-containing protein	*Lilium longiflorum*	LIM1	Promoting filamentous actin bundle assembly	[125]
	*Nicotiana benthamiana*	WLIM1	Promoting the recruitment of actin filaments into thick actin bundles and cables	[126]
NETWORKED protein	*Arabidopsis thaliana*	NET1A	Coupling different membranes to the actin cytoskeleton	[48]
Capping protein	*Arabidopsis thaliana*	CP	Regulating assembly at the barbed ends of actin filaments	[127]

## Data Availability

This is a review article. All data used in this research are included in this article and its supplementary information files.

## Supplementary Materials

The following supporting information can be downloaded at: https://www.mdpi.com/article/10.3390/ijms242015480/s1.
