# Supplementary material for: Exploring the Role of the Plant Actin Cytoskeleton: From Signaling to Cellular Functions
Source: Int J Mol Sci. 2023 Oct 23;24(20):15480. doi: 10.3390/ijms242015480 (PMC10607326; doi:10.3390/ijms242015480)
Supplement: Supplementary file 1 [file ijms-24-15480-s001.zip › ijms-2609009-supplementary.pdf]

Supplementary Material

Supplementary Table S1. Prediction of subcellular localization of actin-related sequences in different plant species.

Species	Prediction	No. of prediction studies per compartment										
		CYT	CSK	NUC	MIT	PLA	PER	ER	GOL	VAC	PM	EXC
Musa acuminata	14	14	0	0	0	0	0	0	0	0	0	0
Hordeum vulgare	1	1	0	0	0	0	0	0	0	0	0	0
Brassica napus	15	15	0	0	0	0	0	0	0	0	0	0
Brassica rapa	6	5	1	0	0	0	0	0	0	0	0	0
Zea mays	39	22	1	3	0	6	0	1	0	0	4	2
Solanum tuberosum	10	9	0	0	1	0	0	0	0	0	0	0
Oryza sativa	2	0	0	0	0	1	0	0	0	0	1	0
Sorghum bicolor	1	1	0	0	0	0	0	0	0	0	0	0
Glycine max	11	3	0	2	3	0	0	3	0	0	0	0
Solanum lycopersicum	5	0	0	3	0	2	0	0	0	0	0	0
Triticum aestivum	3	2	0	0	0	0	0	0	1	0	0	0
Vitis vinifera	2	1	0	0	0	0	0	0	0	0	0	1
Total no. of prediction studies/compartment		73	2	8	4	9	0	4	1	0	5	3

The subcellular localization of proteins highly homologous to *Arabidopsis thaliana* ACT1 (AT2G37620) in other plant species were predicted. The number of distinct prediction studies per species and compartment are shown. The prediction data were obtained from fluorescent protein

(FP), tandem mass spectrometry (MSMS) and related papers in the cropPAL2020 database (cropPAL, https://croppal.org/). This platform allows the search for location data across all crop species as well as compares it to Arabidopsis data from SUBA4 (https://suba.live). The total number of studies per species, methodology and compartment were determined for reference. CYT, cytosol; CSK, cytoskeleton; ER, endoplasmic reticulum; EXC, extracellular; GOL, Golgi; MIT, mitochondrion; NUC, nucleus; PER, peroxisome; PLA, plastid; PM, plasma membrane; VAC, vacuole.

Supplementary Table S2. Prediction of Arabidopsis actin protein subcellular location.

AGI	Predictions	FP	MS/MS	PPI
AT2G37620	mitochondrion<br>cytosol<br>cytoskeleton<br>nucleus	unclear	cytoskeleton<br>cytosol<br>extracellular<br>Golgi (5x)<br>nucleus (2x)<br>plasma membrane (3x)<br>plastid<br>vacuole	AT2G23420<br>AT2G37620<br>AT3G18780<br>AT3G53750
AT3G18780	cytoskeleton<br>mitochondrion<br>nucleus<br>cytosol	vacuole	cytosol<br>Golgi (5x)<br>mitochondrion<br>nucleus (2x)<br>plasma membrane<br>plastid (2x)	AT3G46520<br>AT4G29130<br>AT4G29130<br>AT5G59880
AT3G53750	cytoskeleton<br>mitochondrion<br>nucleus<br>cytosol	unclear	extracellular<br>mitochondrion<br>nucleus	AT2G31200
AT5G59370	cytoskeleton<br>mitochondrion<br>nucleus<br>cytosol	unclear	mitochondrion (2x)	
AT5G09810	cytosol<br>cytoskeleton<br>nucleus<br>mitochondrion	unclear	cytosol<br>Golgi (5x)<br>mitochondrion (2x)<br>nucleus (3x)<br>plasma membrane (9x)<br>plastid (3x)<br>vacuole (2x)	AT2G31200<br>AT2G37620<br>AT3G12110<br>AT3G18060<br>AT5G09810
AT1G49240	nucleus<br>cytoskeleton<br>mitochondrion<br>cytosol	unclear	cytosol (2x)<br>extracellular<br>Golgi (3x)<br>nucleus (2x)<br>peroxisome<br>plasma membrane (5x)<br>plastid (4x)<br>vacuole (2x)	AT1G72770

Supplementary Table S2. (continued). Prediction of Arabidopsis actin protein subcellular location.

AGI	Predictions	FP	MS/MS	PPI
AT3G12110	nucleus<br>cytoskeleton<br>mitochondrion<br>cytosol	unclear	cytoskeleton<br>Golgi (3x)<br>mitochondrion (2x)<br>nucleus<br>plasma membrane (3x)<br>plastid (2x)<br>vacuole	AT3G18780
AT3G46520	cytoskeleton<br>mitochondrion<br>nucleus<br>cytosol	unclear	mitochondrion<br>nucleus<br>plasma membrane (2x)<br>plastid (3x)	AT2G31200<br>AT3G18060<br>AT5G09810

SUBA (https://suba.live) provides a subcellular data query platform, protein sequence BLAST alignment, high-confidence subcellular location reference standards, and analytic tools. Concrete data are collected from the query results of actin proteins on the SUBA platform. AGI, Arabidopsis gene identifier; Predictions, Location prediction summary. Any locations predicted by at least one predictor is shown; FP, Localization summary as determined by fluorescent protein assay; PPI, Protein-protein interactions and the SUBAcon location classification of each interacting protein; MS/MS, Localization summary as determined by mass spectrometry. The values in parentheses represent the intensities of the main fragment ions.
